# Incidence of and Factors Associated With Leprosy Among Household Contacts of Patients With Leprosy in Brazil

**DOI:** 10.1001/jamadermatol.2020.0653

**Published:** 2020-04-15

**Authors:** Camila Silveira Silva Teixeira, Júlia Moreira Pescarini, Flávia Jôse Oliveira Alves, Joilda Silva Nery, Mauro Niskier Sanchez, Carlos Teles, Maria Yury Travassos Ichihara, Anna Ramond, Liam Smeeth, Maria Lucia Fernandes Penna, Laura Cunha Rodrigues, Elizabeth B. Brickley, Gerson Oliveira Penna, Maurício Lima Barreto, Rita de Cássia Ribeiro Silva

**Affiliations:** 1 Centro de Integração de Dados e Conhecimentos para Saúde, Fundação Oswaldo Cruz, Salvador, Brazil; 2 Instituto de Saúde Coletiva, Universidade Federal da Bahia, Salvador, Brazil; 3 Núcleo de Medicina Tropical, Universidade de Brasília, Brasília, Brazil; 4 Department of Infectious Disease Epidemiology, London School of Hygiene & Tropical Medicine, London, United Kingdom; 5 Department of Non-communicable Disease Epidemiology, London School of Hygiene & Tropical Medicine, London, United Kingdom; 6 Health Data Research, London, United Kingdom; 7 Departamento de Epidemiologia e Bioestatística, Universidade Federal Fluminense, Rio de Janeiro, Brazil; 8 Escola Fiocruz do Governo, Fiocruz Brasília, Brasília, Brazil; 9 Escola de Nutrição, Universidade Federal da Bahia, Salvador, Brazil

## Abstract

**Importance:**

Despite progress toward reducing global incidence, leprosy control remains a challenge in low- and middle-income countries.

**Objective:**

To estimate new case detection rates of leprosy among household contacts of patients with previously diagnosed leprosy and to investigate its associated risk factors.

**Design, Setting, and Participants:**

This population-based cohort study included families registered in the 100 Million Brazilian Cohort linked with nationwide registries of leprosy; data were collected from January 1, 2007, through December 31, 2014. Household contacts of patients with a previous diagnosis of leprosy from each household unit were followed up from the time of detection of the primary case to the time of detection of a subsequent case or until December 31, 2014. Data analysis was performed from May to December 2018.

**Exposures:**

Clinical characteristics of the primary case and sociodemographic factors of the household contact.

**Main Outcomes and Measures:**

Incidence of leprosy, estimated as the new case detection rate of leprosy per 100 000 household contacts at risk (person-years at risk). The association between occurrence of a subsequent leprosy case and the exposure risk factors was assessed using multilevel mixed-effects logistic regressions allowing for state- and household-specific random effects.

**Results:**

Among 42 725 household contacts (22 449 [52.5%] female; mean [SD] age, 22.4 [18.5] years) of 17 876 patients detected with leprosy, the new case detection rate of leprosy was 636.3 (95% CI, 594.4-681.1) per 100 000 person-years at risk overall and 521.9 (95% CI, 466.3-584.1) per 100 000 person-years at risk among children younger than 15 years. Household contacts of patients with multibacillary leprosy had higher odds of developing leprosy (adjusted odds ratio [OR]_,_ 1.48; 95% CI, 1.17-1.88), and the odds increased among contacts aged 50 years or older (adjusted OR, 3.11; 95% CI, 2.03-4.76). Leprosy detection was negatively associated with illiterate or preschool educational level (adjusted OR, 0.59; 95% CI, 0.38-0.92). For children, the odds were increased among boys (adjusted OR, 1.70; 95% CI, 1.20-2.42).

**Conclusions and Relevance:**

The findings in this Brazilian population-based cohort study suggest that the household contacts of patients with leprosy may have increased risk of leprosy, especially in households with existing multibacillary cases and older contacts. Public health interventions, such as contact screening, that specifically target this population appear to be needed.

## Key Points

**Question:**

What are the incidence of and the factors associated with leprosy among household contacts of patients with leprosy in the low-income population of Brazil?

**Findings:**

In this cohort study of data from the 100 Million Brazilian Cohort, the incidence of leprosy among 42 725 household contacts of patients with leprosy was higher than that in the overall cohort and the incidence recorded in 2017 in Brazil. Detection of leprosy was associated with the clinical characteristics of the primary leprosy case.

**Meaning:**

The findings suggest that household contacts of patients with previously diagnosed leprosy should be targeted for public health intervention.

## Teaser

This cohort study evaluates the incidence of leprosy among household contacts of patients with leprosy in Brazil and the factors associated with development of leprosy in these contacts.

## Introduction

Leprosy, which is caused mainly by *Mycobacterium leprae*, persists in populations in low- and middle-income countries.^1^ Current evidence suggests that, within these settings, household contacts of existing patients with leprosy are at high risk for developing leprosy.^2,3,4^ The increased incidence of leprosy in household contacts is likely associated with a combination of increased exposure to infectious cases (eg, contacts of patients with multibacillary leprosy have a 5- to 10-times greater risk of developing leprosy than the general population^4,5^) and the sharing of social risk factors within a given family (eg, lower familial income and unfavorable household living conditions).^5,6,7,8^ To enhance understanding of household leprosy transmission, this study used linked data from the 100 Million Brazilian Cohort to estimate the incidence of leprosy among household contacts of patients with leprosy and to compare the odds of leprosy detection among contacts by potential clinical, geographic, and socioeconomic risk factors.

## Methods

### Study Design and Data Source

In this cohort study, household contacts of patients with leprosy were followed up from January 1, 2007, to December 31, 2014, using geographic and socioeconomic data from the baseline of the 100 Million Brazilian Cohort^9^ (2001-2015) linked with leprosy records from the Notifiable Diseases Information System *(*Sistema de Informação de Agravos de Notificação, SINAN-leprosy) (2007-2014).^10^ Individual records from the 2 data sets were deterministically linked using 5 identifying variables: name, mother’s name, sex, date of birth, and municipality of residence.^11^ A manual assessment of 10 000 random pairs showed sensitivity of 0.91 (95% CI, 0.90-0.92) and specificity of 0.89 (95% CI, 0.88-0.90).^12^ The study was approved by the ethics committees of the Universidade de Brasilia, Brazil, the Instituto Gonçalo Muniz (Fiocruz), Salvador, Brazil, and the London School of Hygiene & Tropical Medicine, London, United Kingdom. No personally identifiable information was included in the data set used for analysis; thus, informed consent was waived by the committees. Data analyses were performed from May to December 2018.

### Setting and Participants

This study included members of the 100 Million Brazilian Cohort enrolled between January 1, 2007, and December 31, 2014, with at least 1 household member aged 15 years or older. We defined the first new leprosy case detected in each household as the primary case and defined individuals residing in the same household with the primary case as household contacts. We excluded individuals belonging to households (1) without at least 1 leprosy case, (2) without at least 1 household contact free of leprosy at the time of detection of the primary case, and (3) in which the primary case was diagnosed before study entry.

### Outcome

The primary outcome was the detection of subsequent leprosy cases (ie, new leprosy cases detected among household contacts after the primary case) in the overall population and the subgroup of children younger than 15 years. Household contacts were followed up from the detection of the primary case until the detection of a subsequent case or until December 31, 2014. In the subanalysis of children younger than 15 years, children were censored on their 15th birthday.

### Exposures

Geographic exposures included area of residence (rural or urban), Brazilian region, and residence in a leprosy high-burden priority municipality (ie, defined by the Brazilian Ministry of Health as all capitals, municipalities with new case detection rate of more than 20 per 100 000 inhabitants, and municipalities outside geographical risk areas with 50 new cases and at least 5 cases in children).^13^

Socioeconomic and demographic exposures included household conditions (ie, household density, construction material, water supply, waste disposal, and electricity), monthly household per capita income, and individual sociodemographic variables (ie, age, sex, self-identified race/ethnicity, educational level, and work condition). For individuals younger than 18 years, we used the education and employment characteristics of the oldest member of the household as proxy for the household head.

Clinical exposures included the clinical features of the primary case (ie, operational classification, based on the number of skin and nerve injuries [ie, paucibacillary or multibacillary]); grade of disability at diagnosis, estimated by sensory and motor functions of the eyes, hands, and feet (ie, grade 0, 1, or 2); and reaction episodes, acute inflammatory conditions triggered by disease severity (ie, none, type 1, 2, or 1 + 2).^14,15^ The operational classification of the primary case and the sex and age of the household contact were considered to be confounders a priori.

### Statistical Analysis

The incidence of leprosy was estimated as the new case detection rate (hereafter, incidence) per 100 000 household contacts at risk (person-years at risk) overall and within subpopulations (ie, by age group, geographic factors, and clinical characteristics of the primary case). We calculated the cumulative incidence of leprosy by age group (<15 years vs ≥15 years) and according to the clinical classification of the primary case (paucibacillary vs multibacillary) using the Nelson-Aalen estimator.^16,17^ We estimated the Levin population attributable risk of being exposed to a leprosy case within the household using previous leprosy incidence estimates from the 100 Million Brazilian Cohort as a proxy for the unexposed population.^8^

We estimated the crude and adjusted odds ratio (OR) of developing a subsequent leprosy case by the clinical features of the primary case and the socioeconomic and demographic characteristics of the household contact using multilevel mixed-effects logistic regressions allowing for state- and household-specific random effects. Adjusted models were built using a backward selection approach, where we first included all variables with *P* < .20 in the univariate analysis and removed variables one by one, maintaining those with *P* < .05 in the final model. We checked all model adjustments. Because of the high missingness of certain variables (eg, reaction type), univariate analyses were performed for all individuals with data for a given covariate, whereas multivariate analyses used a complete case approach excluding individuals with any missing data.

In sensitivity analyses, we assessed potential residual confounding using a full multilevel mixed-effects logistic model adjusting for all socioeconomic and demographic factors. In addition, to test our assumption that subsequent cases occurring in a short period after the primary case were already infected but had longer incubation periods, we excluded subsequent cases that were detected within 2, 6, and 12 months of the primary case diagnosis date. All analyses were performed using Stata, version 15.1 (StataCorp).

## Results

**Figure 1.**
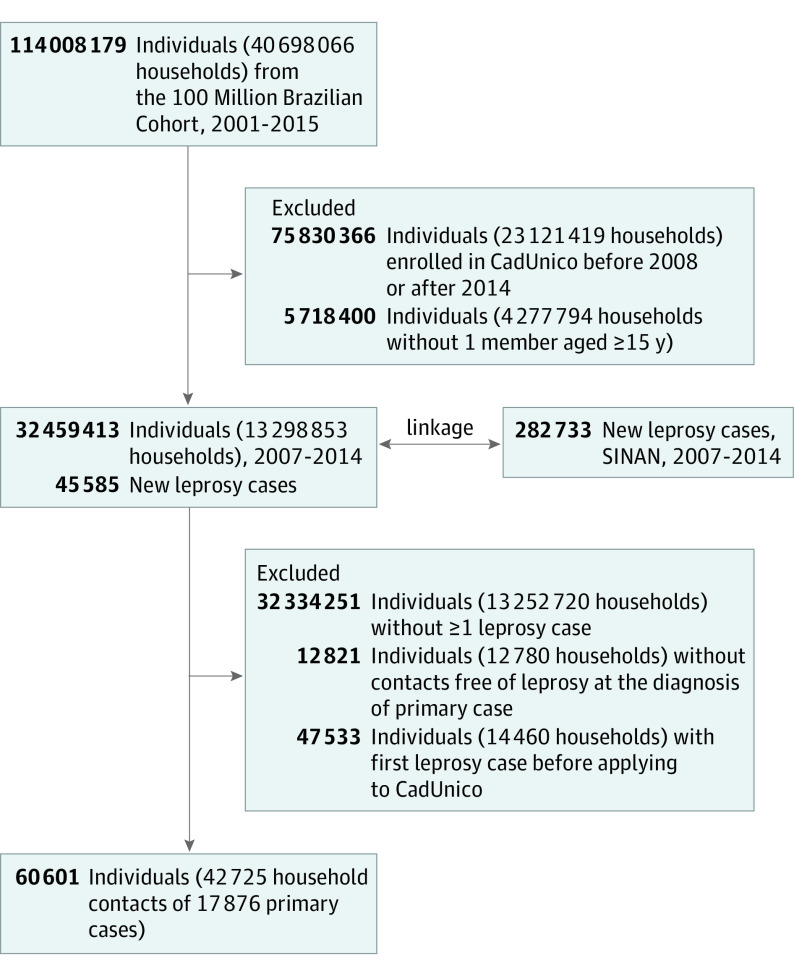
Flowchart CadUnico indicates Cadastro Unico para Programas Sociais; SINAN, Sistema de Informação de Agravos de Notificação.

**Figure 2.**
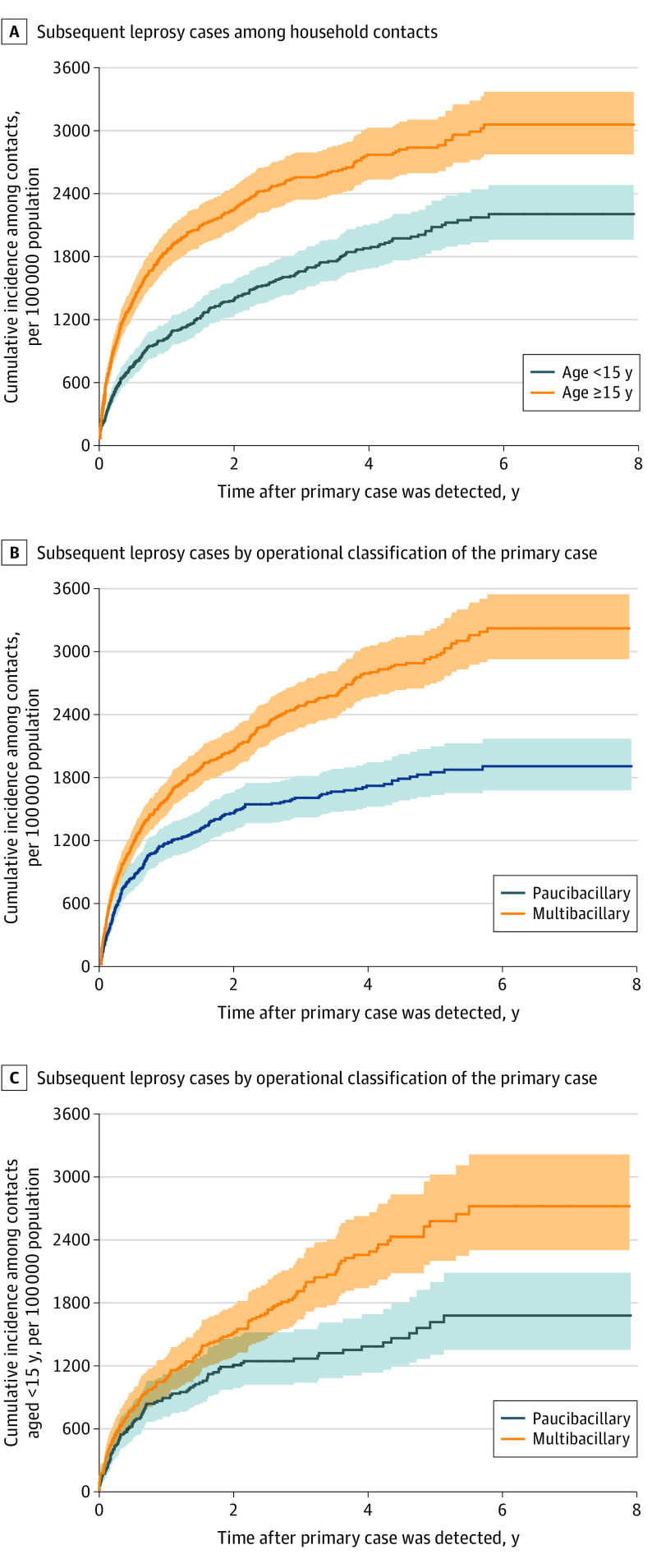
Cumulative Incidence of Subsequent Leprosy Cases Among Households of Patients With Leprosy

The study population included 42 725 household contacts (22 449 [52.5%] female; mean [SD] age, 22.4 [18.5] years) of 17 876 primary cases (Figure 1) followed up for a total of 130 289.3 person-years (median, 2.8 years; interquartile range [IQR], 1.2-4.6 years). We observed 829 subsequent leprosy cases, of which 303 (36.6%) were in children younger than 15 years (Table 1). For both population strata, the detection of subsequent leprosy cases peaked in the first year after detection of the primary case (Figure 2A). The incidence of leprosy among household contacts was 636.3 per 100 000 person-years (95% CI, 594.4-681.1 per 100 000 person-years) overall and 521.9 per 100 000 person-years (95% CI, 466.3-584.1 per 100 000 person-years) among children younger than 15 years. The percentages of cases attributed to exposure inside the household were 97.3% overall and 99.0% among children younger than 15 years. The incidence was broadly consistent across geographic factors (Table 1) and did not vary substantively by socioeconomic factors and living conditions (Table 2).

**Table 1.** Incidence of Leprosy Among Household Contacts by Geographic Factors in the Total Population and Children Younger Than 15 Years

Variable	Household contacts with leprosy, No. (%)	Person-years at risk	Incidence, per 100 000 person-years (95% CI)
Total population (N = 42 725)			
All	829 (1.9)	130 289.3	636.3 (594.4-681.1)
Area of residence^a^			
Urban	631 (1.4)	98 868.0	638.2 (590.3-690.0)
Rural	198 (0.5)	31 253.6	633.5 (551.2-728.2)
Region of residence			
South	20 (0.1)	2657.5	752.6 (485.5-1166.5)
Southeast	110 (0.2)	18 560.5	592.7 (491.6-714.4)
Northeast	288 (0.7)	53 441.2	538.9 (480.1-604.9)
North	177 (0.4)	36 435.9	485.8 (419.2-562.9)
Central-west	234 (0.5)	19 194.2	1219.1 (1072.5-1385.8)
High-burden priority municipalities			
No	444 (1.0)	65 097.6	682.1 (621.5-748.6)
Yes	385 (0.9)	65 191.7	590.6 (534.4-652.4)
Children aged <15 y (n = 20 629)			
All	303 (1.5)	58 060.4	521.9 (466.3-584.1)
Area of residence^a^			
Urban	234 (1.1)	43 048.5	543.6 (478.2-617.9)
Rural	69 (0.4)	14 912.8	462.7 (365.4-585.8)
Region of residence			
South	3 (0)	1035.2	289.8 (92.5-898.5)
Southeast	43 (0.2)	7839.5	548.5 (406.8-739.6)
Northeast	118 (0.6)	23 096.7	510.9 (426.5-611.9)
North	74 (0.4)	17 749.4	416.9 (332.0-523.6)
Central-west	65 (0.3)	8339.5	779.4 (611.2-993.9)
High-burden priority municipalities			
No	150 (0.7)	28 710.6	522.5 (445.2-613.1)
Yes	153 (0.8)	29 349.8	521.3 (444.9-610.8)

^a^ The zone of residence was not recorded for 44 household contacts.

**Table 2.** Household and Individual Characteristics of the Study Population and Incidence of Subsequent Leprosy Cases Among Household Contacts

Characteristic	No. (%)		Incidence, per 100 000 person-years (95%CI)
	Total population (N = 42 725)	Subsequent leprosy cases (n = 829)	
**Household characteristic**			
Per capita income, minimum wage, Brazilian real^a^			
≥0.25	9097 (21.2)	198 (23.9)	834.7 (726.1-959.4)
0-0.24	30 228 (70.8)	566 (68.3)	584.2 (538.0-634.4)
0	3400 (8.0)	65 (7.8)	670.8 (526.1-855.4)
Household density, inhabitants per room			
0-0.9	15 708 (36.8)	324 (39.1)	727.4 (652.4-811.1)
1.00-1.49	14 475 (13.9)	288 (34.7)	632.0 (563.1-709.4)
≥1.50	12 123 (28.3)	213 (25.7)	539.9 (472.1-617.5)
Missing	419 (1.0)	4 (0.5)	NA
Housing construction material			
Bricks or cement	27 812 (65.1)	542 (65.4)	643.4 (591.4-699.9)
Taipa, wood, or other	14 531 (34.0)	283 (34.1)	622.7 (554.3-699.7)
Missing	382 (0.9)	4 (0.5)	NA
Water supply			
Public network	27 491 (64.3)	533 (63.5)	639.6 (587.5-696.3)
Well, natural source, or other	14 852 (34.8)	292 (36.1)	629.9 (561.6-706.4)
Missing	382 (0.9)	4 (0.4)	NA
Waste disposal system			
Public network	12 657 (29.6)	229 (27.6)	589.6 (518.0-671.1)
Septic tank	22 892 (53.6)	474 (57.2)	680.6 (622.0-744.7)
Ditch or other	6333 (14.8)	118 (14.2)	573.5 (478.8-686.9)
Missing	843 (2.0)	8 (1.0)	NA
Electricity supply			
With control meter	34 131 (79.9)	681 (82.2)	658.5 (610.8-709.8)
Without control meter, gas, candlelight, or other	8212 (19.2)	144 (17.4)	548.2 (465.6-645.5)
Missing	417 (0.9)	4 (0.4)	NA
Garbage disposal			
Public collection system	30 849 (72.2)	600 (72.4)	639.6 (590.4-693.9)
Burned, buried, or other	11 494 (26.9)	225 (27.1)	627.0 (550.2-714.6)
Missing	382 (0.9)	4 (0.2)	NA
**Individual characteristic of the contacts**			
Sex			
Female	22 449 (52.5)	436 (52.6)	639.9 (582.6-702.9)
Male	20 276 (47.5)	393 (47.4)	632.3 (572.8-698.0)
Age, y			
<5	5519 (12.9)	69 (8.3)	341.6 (269.8-432.5)
5-9	8194 (19.2)	124 (15.0)	483.0 (405.0-575.9)
10-14	6916 (16.2)	129 (15.6)	625.5 (526.3-743.3)
15-29	9688 (22.7)	161 (19.4)	554.3 (474.9-646.8)
30-49	7899 (18.5)	190 (22.9)	843.3 (731.5-972.1)
≥50	4509 (10.5)	156 (18.8)	1277.3 (1091.8-1494.3)
Race/ethnicity			
White	7631 (17.9)	147 (17.7)	649.9 (552.9-763.9)
Black	2545 (5.9)	61 (7.4)	762.8 (593.5-980.3)
Asian	117 (0.3)	4 (0.5)	1291.6 (484.8-3441.4)
Mixed	31 924 (74.7)	609 (73.5)	620.3 (572.9-671.6)
Indigenous	173 (0.4)	2 (0.2)	382.2 (95.6-1528.3)
Missing	335 (0.8)	6 (0.7)	NA
Educational level			
High school or college	6676 (15.6)	144 (17.4)	683.9 (580.8-805.2)
Elementary or middle school (4-9 y of formal education)	15 295 (35.8)	304 (36.7)	633.6 (566.3-709.0)
Elementary school (<4 y of formal education)	11 398 (26.7)	224 (27.0)	649.2 (569.5-740.0)
Illiterate or preschool	4672 (10.9)	72 (8.7)	599.3 (475.7-755.0)
Missing	4684 (10.9)	85 (10.2)	NA
Work condition			
Employed	21 031 (49.2)	393 (47.4)	598.1 (541.8-660.2)
Unemployed but currently studying	10 847 (25.4)	221 (26.7)	585.7 (513.3-668.2)
Unemployed	8105 (19.0)	158 (19.1)	749.5 (641.3-876.0)
Missing	2742 (6.4)	57 (6.8)	NA

Abbreviation: NA, not applicable.

^a^ Minimum wage was 181 Brazilian real in 2014.

In both the total population and children younger than 15 years, the incidence of leprosy was higher among contacts of patients with multibacillary leprosy, grade-2 physical disabilities, or reactions type 1 + 2 (eTable 1 in the Supplement). The incidence among household contacts of patients with multibacillary leprosy was approximately 60% higher than that among household contacts of patients with paucibacillary leprosy, with similar associations over time (Figure 2B and C and eTable 1 in the Supplement).

After adjusting for sex and age, contacts of patients with multibacillary leprosy had higher odds of having leprosy detected (adjusted OR, 1.48; 95% CI, 1.17-1.88) (Table 3). Contacts aged 50 years or older had more than 3 times the odds of leprosy than children younger than 5 years (adjusted OR, 3.11; 95% CI, 2.03-4.76), and illiterate or preschool-educated contacts had lower leprosy detection compared with individuals attaining high school education (adjusted OR, 0.59; 95% CI, 0.38-0.92). For children younger than 15 years, leprosy detection was also increased among males (adjusted OR, 1.70, 95% CI, 1.20-2.42) (Table 3).

**Table 3.** Odds Ratios for Detecting Subsequent Leprosy Cases Among Household Contacts for the Total Population and Children Younger Than 15 Years

Characteristic	OR (95% CI)			
	Total population		<15 y	
	Unadjusted (N = 42 725)^a^	Adjusted (n = 25 955)^b^^,^^c^	Unadjusted (n = 20 629)^a^	Adjusted (n = 13 403)^b^^,^^c^
**Household characteristic**				
Area of residence				
Urban	1 [Reference]	NA	1 [Reference]	NA
Rural	1.14 (0.92-1.42)	NA	0.90 (0.63-1.27)	NA
Per capita income, minimum wage				
≥0.25	1 [Reference]	NA	1 [Reference]	NA
0.01-0.24	0.95 (0.77-1.18)	NA	1.34 (0.86-2.10)	NA
0	0.92 (0.64-1.32)	NA	1.62 (0.88-2.96)	NA
Household density, inhabitants per room				
0-0.99	1 [Reference]	NA	1 [Reference]	NA
1.00-1.49	1.01 (0.82-1.23)	NA	1.10 (0.77-1.57)	NA
≥1.50	0.92 (0.73-1.16)	NA	1.20 (0.82-1.74)	NA
Housing construction material				
Bricks or cement	1 [Reference]	NA	1 [Reference]	NA
Taipa, wood, or others	1.05 (0.85-1.30)	NA	0.92 (0.68-1.24)	NA
Water supply				
Public network, tap water	1 [Reference]	NA	1 [Reference]	NA
Well, natural source, or others (cisterna or other not described)	1.12 (0.92-1.37)	NA	0.87 (0.64-1.18)	NA
Waste disposal system				
Public network	1 [Reference]	NA	1 [Reference]	NA
Homemade or septic tank	1.09 (0.88-1.36)	NA	1.03 (0.74-1.45)	NA
Ditch or others	1.16 (0.86-1.57)	NA	0.97 (0.61-1.54)	NA
Electricity supply				
With control meter	1 [Reference]	NA	1 [Reference]	NA
Without control meter, gas, candlelight, or others	0.99 (0.78-1.26)	NA	0.80 (0.56-1.16)	NA
Garbage disposal				
Public collection system	1 [Reference]	NA	1 [Reference]	NA
Burned, buried, outdoor disposal, or others	1.10 (0.90-1.36)	NA	0.79 (0.57-1.11)	NA
**Clinical characteristic of the primary case**				
World Health Organization operation classification				
Paucibacillary	1 [Reference]	1 [Reference]	1 [Reference]	1 [Reference]
Multibacillary	1.56 (1.29-1.88)	1.48 (1.17-1.88)	1.50 (1.11-2.04)	1.49 (1.01-2.21)
Physical disability at the diagnosis, grade				
0	1 [Reference]	NA	1 [Reference]	NA
1	1.03 (0.82-1.28)	NA	0.80 (0.54-1.20)	NA
2	1.32 (0.92-1.91)	NA	1.28 (0.69-2.38)	NA
Reaction type				
None	1 [Reference]	NA	1 [Reference]	NA
1	1.04 (0.79-1.38)	NA	1.42 (0.90-2.24)	NA
2	1.41 (0.85-2.35)	NA	1.20 (0.49-2.95)	NA
1 + 2	2.82 (1.49-5.34)	NA	3.45 (1.13-10.51)	NA
**Individual characteristic of the contacts**				
Sex				
Female	1 [Reference]	1 [Reference]	1 [Reference]	1 [Reference]
Male	1.00 (0.86-1.17)	1.13 (0.93-1.38)	1.30 (0.99-1.71)	1.70 (1.20-2.42)
Age, y				
<5	1 [Reference]	1 [Reference]	1 [Reference]	1 [Reference]
5-9	1.24 (0.89-1.73)	1.15 (0.76-1.74)	1.34 (0.87-1.75)	1.09 (0.71-1.69)
10-14	1.70 (1.21-2.37)	1.44 (0.95-2.19)	1.41 (0.98-2.02)	1.20 (0.77-1.90)
15-29	1.52 (1.10-2.08)	1.57 (1.06-2.34)	NA	NA
30-49	2.32 (1.69-3.18)	2.42 (1.63-3.59)	NA	NA
≥50	3.55 (2.54-5.00)	3.11 (2.03-4.76)	NA	NA
Race/ethnicity				
White	1 [Reference]	NA	1 [Reference]	NA
Not white	1.12 (0.90-1.41)	NA	1.49 (0.98-2.25)	NA
Schooling				
High school or college	1 [Reference]	1 [Reference]	1 [Reference]	NA
Elementary or middle school (4-9 y of formal education)	0.89 (0.69-1.15)	0.86 (0.63-1.16)	0.79 (0.51-1.23)	NA
Elementary school (<4 y of formal education)	0.84 (0.64-1.10)	0.96 (0.70-1.33)	1.09 (0.70-1.70)	NA
Illiterate or preschool	0.65 (0.46-0.92)	0.59 (0.38-0.92)	0.76 (0.41-1.39)	NA
Work condition				
Employed	1 [Reference]	NA	1 [Reference]	NA
Unemployed but currently studying	1.18 (0.96-1.44)	NA	0.91 (0.63-1.33)	NA
Unemployed	1.08 (0.87-1.36)	NA	0.77 (0.50-1.20)	NA

^a^ Univariate multilevel logistic regression model accounting for household and state-level random effects.

^b^ Final model of multilevel logistic regression accounting for household and state-level random effects with a priori adjustment for operational classification of the primary case and sex and age of the contact and exclusion of individuals with missing data.

^c^ For all the tests and for inclusion of the variables in the final model, a significance level of 5% was used. Multivariate models were created using a backward selection approach and evaluated using the Akaike information criterion. The goodness of fit of the final model was also assessed.

In the sensitivity analyses, full-adjusted models were similar to the primary analysis (eTable 2 in the Supplement). After exclusion of subsequent cases diagnosed within 2, 6, and 12 months of the primary case, leprosy cases detected later in time were more likely to be associated with being a contact of a patient with multibacillary leprosy and with having a high school or college education (eTable 3 in the Supplement). For children, leprosy cases detected later in time were associated with being a contact of a patient with multibacillary leprosy, being younger (age 0-5 years), and being male (eTable 4 in the Supplement).

## Discussion

In conducting a nationwide analysis of 42 725 household contacts of leprosy cases from the 100 Million Brazilian Cohort, this investigation provided robust estimates of the incidence of leprosy among household contacts. Among these contacts, leprosy incidence was estimated to be approximately 37-times higher than that in the 100 Million Brazilian Cohort overall (17.1 per 100 000 person-years)^8^ and 50-times higher than the rate recorded for the general population of Brazil in 2017 (12.9 per 100 000 person-years).^18^ Furthermore, although household contacts younger than 15 years had a lower detection rate of leprosy than adults, the rate was 100 times higher than in the full population of children from the 100 Million Brazilian Cohort (5.2 per 100 000 person-years).^8^ Overall, these results were similar to previously reported new case detection rates of 80 per 100 000 person-years,^4^ 364 per 100 000 person-years,^3^ and 676 per 100 000 person-years^19^ among household contacts in China, Malawi, and India. Together, these findings suggest that there is a high incidence of leprosy among household contacts compared with individuals with similar low-income status.

Within the total population, individuals who resided with patients with multibacillary leprosy, were aged 50 years or older, or had attained at least a high school educational level had increased odds of leprosy detection. In contrast, other geographic, socioeconomic, and individual-level characteristics that have previously been shown to be associated with an increased risk of leprosy detection^8^ were not associated with leprosy detection among household contacts. These findings suggest that the risk associated with living in increased proximity to a primary leprosy case may supersede individual-level and geographic leprosy risk factors for becoming a subsequent leprosy case.

Higher leprosy rates among household contacts of patients with multibacillary leprosy might be explained by the exposure to relatively higher bacillary load.^20,21^ Similar to our findings, previous research has reported higher odds of leprosy detection among contacts who are older^5,22,23^ and male.^2^ In this study, we found lower leprosy detection among contacts with lower educational levels. However, it is plausible that after a primary leprosy case in the household, contacts with education beyond the preschool level may have had improved leprosy knowledge, increased health-seeking behavior, and/or better access to health services that may have enhanced their case detection rates.^24^

Social development has been central to leprosy control historically^25^ and remains key to reducing leprosy burden in contacts as well as in the general population. In this study, leprosy risk among household contacts was similar across geographic location or socioeconomic conditions of households, which differed from previous studies.^8,25,26^ However, given that the households affected by leprosy in the 100 Million Brazilian Cohort were more likely to have low-income circumstances,^8^ the sample in the present study was relatively homogeneously composed of individuals of limited resources, which may have limited our ability to differentiate any health outcomes associated with socioeconomic status.^27^

The high proportion of cases associated with exposure to leprosy cases within the household compared with exposure outside of household suggests that household contacts with low-income status may benefit from targeted and effective strategies to prevent transmission, such as strengthening screening of contacts. Although immunotherapy and chemoprophylaxis remain a challenge,^28^ the dermatoneurological examination of household contacts continues to be the criterion standard approach for mitigating risks to household contacts. In 2017, a total of 78.9% of contacts of patients with leprosy were examined across Brazil.^18^ Since the *Global Leprosy Strategy 2016-2020*,^15^ national guidelines have been expanded for surveillance of social contacts, but their implementation is still restricted because of the stigma associated with the disease and, in some regions, the lack of trained health care professionals. The training of professionals to screen contacts and health education (eg, pamphlets, lectures, and screening campaigns) will continue to be important strategies for detecting leprosy early, reducing stigmatizing disabilities, and preventing subsequent transmission.

### Limitations

Although this study has provided a unique opportunity to investigate leprosy in a large cohort of household contacts from national health- and administrative-linked databases, it also has limitations. In relying on routinely collected records, the data set had a considerable proportion of missingness for certain variables and also unmeasured confounders, such as health-seeking behavior and proximity to health services. In addition, because the proportion of households of patients with leprosy evaluated in Brazil is still insufficient (<80%)^18^ and leprosy reporting to the SINAN system is passive, this study may underestimate the true incidence of leprosy among household contacts. Also, because the population of the 100 Million Brazilian Cohort consists of applicants to social programs, the findings may not be generalizable to all household contacts of patients with leprosy in Brazil.

## Conclusions

The findings suggest that household contacts of patients with leprosy may have increased risk of leprosy, especially in households with existing multibacillary cases and older contacts. Strengthening public health interventions, such as contact screening, along with social interventions that specifically target this population appear to be needed.

## Notes

Supplement. eTable 1. Incidence of Subsequent Leprosy Cases Among Household Contacts by Clinical Characteristics of the Primary Cases for the Total Population and Children Under 15 Years in the 100 Million Brazilian Cohort, 2007-2014 eTable 2. Incidence of Subsequent Leprosy Cases Among Household Contacts for the Total Population and Children Under 15 Years Adjusted by a Full Multilevel Logistic Regression Model in the 100 Million Brazilian Cohort, 2007-2014 eTable 3. Incidence of Leprosy for Not Co-prevalent Subsequent Cases Diagnosed Among Household Contacts (Diagnostic Date Over 2, 6, and 12 Months After Primary Case) in the 100 Million Brazilian Cohort, 2007-2014 eTable 4. Incidence of Leprosy for Not Co-prevalent Subsequent Cases Diagnosed Among Children Household Contacts Under 15 Years (Diagnostic Date Over 2, 6, and 12 Months After Primary Case). The 100 Million Brazilian Cohort, 2007-2014.
