# Supplementary material for: Incidence of and Factors Associated With Leprosy Among Household Contacts of Patients With Leprosy in Brazil
Source: JAMA Dermatol. 2020 Apr 15;156(6):1–10. doi: 10.1001/jamadermatol.2020.0653 (PMC7160739; doi:10.1001/jamadermatol.2020.0653)
Supplement: Supplement. — eTable 1. Incidence of Subsequent Leprosy Cases Among Household Contacts by Clinical Characteristics of the Primary Cases for the Total Population and Children Under 15 Years in the 100 Million Brazilian Cohort, 2007-2014 eTable 2. Incidence of Subsequent Leprosy Cases Among Household Contacts for the Total Population and Children Under 15 Years Adjusted by a Full Multilevel Logistic Regression Model in the 100 Million Brazilian Cohort, 2007-2014 eTable 3. Incidence of Leprosy for Not Co-prevalent Subsequent Cases Diagnosed Among Household Contacts (Diagnostic Date Over 2, 6, and 12 Months After Primary Case) in the 100 Million Brazilian Cohort, 2007-2014 eTable 4. Incidence of Leprosy for Not Co-prevalent Subsequent Cases Diagnosed Among Children Household Contacts Under 15 Years (Diagnostic Date Over 2, 6, and 12 Months After Primary Case). The 100 Million Brazilian Cohort, 2007-2014. [file jamadermatol-156-640-s001.pdf]

Supplementary Online Content

Teixeira CSS, Pescarini JM, Alves FJO, et al. Incidence of and factors associated with leprosy among household contacts of patients with leprosy Brazil. *JAMA Dermatol*. Published online April 15, 2020. doi:10.1001/jamadermatol.2020.0653

eTable 1. Incidence of Subsequent Leprosy Cases Among Household Contacts by Clinical Characteristics of the Primary Cases for the Total Population and Children Under 15 Years in the 100 Million Brazilian Cohort, 2007-2014.

eTable 2. Incidence of Subsequent Leprosy Cases Among Household Contacts for the Total Population and Children Under 15 Years Adjusted by a Full Multilevel Logistic Regression Model in the 100 Million Brazilian Cohort, 2007-2014.

eTable 3. Incidence of Leprosy for Not Co-prevalent Subsequent Cases Diagnosed Among Household Contacts (Diagnostic Date Over 2, 6, and 12 Months After Primary Case) in the 100 Million Brazilian Cohort, 2007-2014.

eTable 4. Incidence of Leprosy for Not Co-prevalent Subsequent Cases Diagnosed Among Children Household Contacts Under 15 Years (Diagnostic Date Over 2, 6, and 12 Months After Primary Case). The 100 Million Brazilian Cohort, 2007-2014.

This supplementary material has been provided by the authors to give readers additional information about their work.

© 2020 Teixeira CSS et al. *JAMA Dermatology.*

Table 1. Incidence of Subsequent Leprosy Cases Among Household Contacts by Clinical Characteristics of the Primary Cases for the Total Population and Children Under 15 Years in the 100 Million Brazilian Cohort, 2007-2014

**Clinical characteristics of the primary case**	**Subsequent leprosy cases**	**Person-years at risk**	**Incidence per 100,000 person-years**	**95%CI**
**Total population (N=42,725)**	829	130,289.3	636.3	594.4 – 681.1
WHO operational classification[1]				
Paucibacillary	268	57,654.3	464.8	412.4 – 524.0
Multibacillary	560	72,529.2	772.1	710.7 – 838.8
Physical disability at the diagnosis [2]				
Grade 0	523	84,427.0	619.5	568.6 – 674.9
Grade 1	171	26,426.7	647.1	557.0 – 751.7
Grade 2	59	6,923.7	852.1	660.2 – 1,099.8
Reaction episode[3]				
No reaction	543	82,559.7	657.7	604.6 – 715.4
Reaction type 1	100	14,553.3	687.1	564.8 – 835.9
Reaction type 2	29	3,494.9	829.8	576.6 – 1,194.1
Reaction type 1+2	19	1,370.8	1,386.1	884.1 – 2,173.0
**Children <15 years (N=20,629)**	303	58,060.4	521.9	466.3 – 584.1
WHO operational classification[1]				
Paucibacillary	106	26,415.1	401.3	331.7 – 485.4
Multibacillary	197	31,601.9	623.4	542.1 – 716.8
Physical disability at the diagnosis [2]				
Grade 0	200	38,120.7	524.6	456.8 – 602.6
Grade 1	51	11,441.9	445.7	338.7 – 586.5
Grade 2	21	2,938.6	714.6	465.9 – 1,096.0
Reaction episode[3]				
No reaction	190	36,949.8	514.2	446.1 – 592.8
Reaction type 1	45	6,459.2	696.7	520.2 – 933.1
Reaction type 2	9	1,577.2	570.6	296.9 – 1,096.7
Reaction type 1+2	7	584.2	1,198.3	571.3 – 2,513.5

[1]For 37 household contacts the operational classification of the primary case was not recorded.

[2]For 4,095 household contacts the grade of disability at diagnosis of the primary case was not recorded.

[3]For 8,919 household contacts the occurrence of reaction episode of the primary case was not recorded.

© 2020 Teixeira CSS et al. *JAMA Dermatology.*

Table 2. Incidence of Subsequent Leprosy Cases Among Household Contacts for the Total Population and Children Under 15 Years Adjusted by a Full Multilevel Logistic Regression Model in the 100 Million Brazilian Cohort, 2007-2014

**Variables**	**Total population**	**Under 15 years**
	**OR_{adj}[1] (95%CI) N= 25,180**	**OR_{adj}[1] (95%CI) N= 12,385**
**Household characteristics**		
Area of residence		
Urban	1.00	1.00
Rural	1.06 (0.73-1.56)	1.01 (0.53-1.93)
Per capita income		
≥0.25 minimum wage	1.00	1.00
0.01 - 0.24 minimum wage	1.29 (0.94-1.77)	1.13 (0.61-2.07)
0 minimum wage	1.33 (0.81-2.19)	1.85 (0.77-4.47)
Household density		
0 - 0.99 inhab/room	1.00	1.00
1.0 - 1.49 inhab/room	1.31 (0.99-1.74)	1.29 (0.78-2.12)
≥1.5 inhab/room	1.17 (0.85-1.62)	1.61 (0.94-2.76)
Housing construction material		
Bricks/Cement	1.00	1.00
Taipa/Wood/Others	0.97 (0.72-1.32)	0.85 (0.52-1.38)
Water supply		
Public network (tap water)	1.00	1.00
Well/natural source/Others (cisterna or other not described)	1.09 (0.81-1.49)	0.88 (0.54-1.46)
Waste disposal system		
Public network	1.00	1.00
Homemade or Septic tank	1.03 (0.76-1.39)	1.17 (0.71-1.92)
Ditch/Others	1.05 (0.67-1.65)	1.12 (0.53-2.36)
Electricity supply		
Electricity with counter	1.00	1.00
Electricity without counter/ Gas/Candlelight/Others	0.82 (0.58-1.16)	0.83 (0.47-1.44)
Garbage disposal		
Public collection system	1.00	1.00
Burned/buried/outdoor disposal/others	1.08 (0.73-1.60)	0.95 (0.49-1.84)
**Clinical characteristics of the primary case**		
WHO operation classification		
Paucibacillary	1.00	1.00
Multibacillary	1.52 (1.16-1.97)	1.52 (0.98-2.36)
Physical disability at the diagnosis		
Grade 0	1.00	1.00
Grade 1	0.94 (0.71-1.25)	0.82 (0.49-1.37)
Grade 2	1.33 (0.86-2.06)	1.35 (0.63-2.89)
Reaction episode		
No reaction	1.00	1.00
Reaction type 1	0.93 (0.66-1.30)	1.12 (0.64-1.97)
Reaction type 2	1.13 (0.61-2.11)	1.25 (0.44-3.50)

© 2020 Teixeira CSS et al. *JAMA Dermatology.*

Reaction type 1 + 2	2.04 (0.92-4.52)	1.86 (0.44-7.87)
**Individual characteristics of the contacts**		
Sex		
Female	1.00	1.00
Male	1.16 (0.95-1.42)	1.70 (1.19-2.44)
Age		
<5 years	1.00	1.00
5 - 9 years	1.10 (0.72-1.67)	1.07 (0.68-1.68)
10 - 14 years	1.38 (0.90-2.10)	1.09 (0.68-1.75)
15 - 29 years	1.46 (0.97-2.19)	-
30 - 49 years	2.36 (1.58-3.53)	-
≥ 50 years	3.44 (2.20-5.39)	-
Race/Ethnicity		
White	1.00	1.00
Not white	1.23 (0.92-1.64)	1.59 (0.93-2.74)
Schooling		
High school/College	1.00	1.00
Elementary/Middle school (4 - 9 years of formal education)	0.80 (0.56-1.14)	0.65 (0.35-1.22)
Elementary school (<4 years of formal education)	0.90 (0.62-1.32)	0.99 (0.52-1.89)
Illiterate/preschool	0.63 (0.39-1.01)	0.61 (0.26-1.42)
Work condition		
Employed	1.00	1.00
Unemployed but currently studying	1.11 (0.85-1.44)	0.82 (0.49-1.39)
Unemployed	0.94 (0.66-1.34)	0.64 (0.31-1.30)

[1]Full model of multilevel logistic regression accounting for household and state levels using random effects.

© 2020 Teixeira CSS et al. *JAMA Dermatology.*

Table 3. Incidence of Leprosy for Not Co-prevalent Subsequent Cases Diagnosed Among Household Contacts (Diagnostic Date Over 2, 6, and 12 Months After Primary Case) in the 100 Million Brazilian Cohort, 2007-2014

**Variables**	**Subsequent cases with diagnostic date >2 months (N=613)**		**Subsequent cases with diagnostic date >6 months (N=438)**		**Subsequent cases with diagnostic date >12 months (N=304)**	
	**OR[1] (95%CI)**	**OR_{adj}[2,3] (95%CI)**	**OR[1] (95%CI)**	**OR_{adj}[2,3] (95%CI)**	**OR[1] (95%CI)**	**OR_{adj}[2,3] (95%CI)**
	**(N=25,787)**	**(N=25,787)**	**(N=42,284)**	**(N=25,661)**	**(N=42,123)**	**(N=25,567)**
**Household characteristics**						
Area of residence						
Urban	1.00		1.00		1.00	
Rural	1.15 (0.91-1.46)		1.12 (0.84-1.48)		1.02 (0.72-1.44)	
Per capita income						
≥0.25 minimum wage	1.00		1.00		1.00	
0 - 0.24 minimum wage	1.12 (0.87-1.44)		1.25 (0.92-1.69)		1.40 (0.96-2.04)	
0 minimum wage	1.00 (0.66-1.52)		0.99 (0.60-1.65)		0.78 (0.39-1.56)	
Household density						
0 - 0.99 inhab/room	1.00		1.00		1.00	
1.0 - 1.49 inhab/room	1.13 (0.89-1.42)		1.22 (0.92-1.61)		1.15 (0.81-1.63)	
≥1.5 inhab/room	1.03 (0.80-1.34)		1.27 (0.94-1.72)		1.49 (1.04-2.13)	
Housing construction material						
Bricks/cement	1.00		1.00		1.00	
Taipa/Wood/Others	1.22 (0.97-1.55)		1.22 (0.93-1.61)		1.22 (0.88-1.67)	
Water supply						
Public network (tap water)	1.00		1.00		1.00	
Well/natural source/Others	1.22 (0.98-1.52)		1.28 (0.97-1.62)		1.17 (0.86-1.58)	

© 2020 Teixeira CSS et al. *JAMA Dermatology.*

(cisterna or other not described)						
Waste disposal system						
Public network	1.00		1.00		1.00	
Homemade or Septic tank	1.14 (0.89-1.47)		1.20 (0.89-1.60)		1.04 (0.74-1.47)	
Ditch/Others	1.40 (1.00-1.95)		1.31 (0.89-1.94)		1.44 (0.93-2.24)	
Electricity supply						
Electricity with counter	1.00		1.00		1.00	
Electricity without counter/ Gas/Candlelight/Others	1.15 (0.89-1.49)		1.22 (0.91-1.65)		1.25 (0.88-1.78)	
Garbage disposal						
Public collection system	1.00		1.00		1.00	
Burned/buried/Outdoor disposal/ Others	1.12 (0.88-1.41)		1.19 (0.90-1.55)		1.16 (0.84-1.60)	
**Clinical characteristics of the primary case**						
WHO operation classification						
Paucibacillary	1.00	1.00	1.00	1.00	1.00	1.00
Multibacillary	1.53 (1.24-1.89)	1.51 (1.15-1.98)	1.71 (1.33-2.21)	1.73 (1.24-2.41)	1.96 (1.43-2.68)	2.43 (1.60-3.69)
Physical disability at the diagnosis						
Grade 0	1.00		1.00		1.00	
Grade 1	1.08 (0.84-1.38)		1.09 (0.81-1.47)		1.33 (0.94-1.90)	
Grade 2	1.16 (0.76-1.77)		1.21 (0.74-1.99)		1.53 (0.87-2.70)	
Reaction episode						
No reaction	1.00		1.00		1.00	
Reaction type 1	1.11 (0.81-1.53)		1.14 (0.78-1.66)		1.23 (0.79-1.92)	
Reaction type 2	1.88 (1.10-3.21)		1.60 (0.82-3.11)		1.97 (0.93-4.16)	
Reaction type 1 + 2	2.18 (1.00-4.75)		3.09 (1.34-7.09)		3.19 (1.20-8.44)	

© 2020 Teixeira CSS et al. *JAMA Dermatology.*

**Individual characteristics of the contacts**						
Sex						
Female	1.00	1.00	1.00	1.00	1.00	1.00
Male	0.93 (0.78-1.11)	1.06 (0.84-1.34)	0.89 (0.72-1.11)	1.08 (0.82-1.43)	0.98 (0.75-1.28)	1.11 (0.79-1.55)
Age						
<5 years	1.00	1.00	1.00	1.00	1.00	1.00
5 - 9 years	1.08 (0.76-1.53)	0.97 (0.63-1.49)	0.88 (0.59-1.30)	0.79 (0.49-1.28)	0.84 (0.53-1.34)	0.65 (0.37-1.13)
10 - 14 years	1.15 (0.80-1.66)	0.81 (0.51-1.30)	0.92 (0.61-1.40)	0.59 (0.34-1.01)	0.98 (0.61-1.58)	0.58 (0.32-1.04)
15 - 29 years	1.11 (0.79-1.56)	1.05 (0.69-1.62)	0.87 (0.59-1.28)	0.76 (0.47-1.23)	0.76 (0.48-1.21)	0.58 (0.33-1.01)
30 - 49 years	1.61 (1.15-2.27)	1.48 (0.97-2.28)	1.19 (0.81-1.74)	1.05 (0.65-1.72)	0.83 (0.51-1.34)	0.63 (0.35-1.14)
≥50 years	2.45 (1.71-3.53)	1.97 (1.25-3.11)	1.72 (1.14-2.61)	1.33 (0.79-2.25)	1.51 (0.92-2.48)	0.91 (0.49-1.68)
Race/Ethnicity						
White	1.00		1.00		1.00	
Not White	1.14 (0.88-1.48)		1.13 (0.83-1.53)		1.46 (0.97-2.18)	
Schooling						
High school/College	1.00	1.00	1.00	1.00	1.00	1.00
Elementary/Middle school (4 - 9 years of formal education)	0.76 (0.57-1.01)	0.68 (0.49-0.95)	0.65 (0.47-0.90)	0.57 (0.39-0.84)	0.71 (0.48-1.05)	0.65 (0.41-1.03)
Elementary school (< 4 years of formal education)	0.77 (0.57-1.03)	0.83 (0.58-1.19)	0.72 (0.51-1.01)	0.75 (0.50-1.13)	0.71 (0.47-1.08)	0.78 (0.48-1.28)
Illiterate/preschool	0.45 (0.30-0.69)	0.31 (0.17-0.54)	0.36 (0.22-0.61)	0.22 (0.10-0.45)	0.32 (0.17-0.63)	0.21 (0.08-0.54)
Work condition						
Employed	1.00		1.00		1.00	
Unemployed but currently studying	1.09 (0.86-1.38)		1.14 (0.87-1.53)		1.03 (0.74-1.42)	
Unemployed	1.11 (0.87-1.43)		1.14 (0.84-1.50)		0.70 (0.47-1.05)	

[1]Univariate multilevel logistic regression model accounting for household and state level random effects.

© 2020 Teixeira CSS et al. *JAMA Dermatology.*

[2]Final model of multilevel logistic regression accounting for household and state level random effects with a priori adjustment for operational classification of the primary case, and sex and age of the contact and exclusion of individuals with missing data.

[3]For all the tests and for inclusion of the variables in the final model was used a significance level of 5%. Multivariate models were created using a backward selection approach and evaluated using the Akaike Information Criterion. The goodness of fit of the final model was also assessed.

© 2020 Teixeira CSS et al. *JAMA Dermatology.*

Table 4. Incidence of Leprosy for Not Co-prevalent Subsequent Cases Diagnosed Among Children Household Contacts Under 15 Years (Diagnostic Date Over 2, 6, and 12 Months After Primary Case). The 100 Million Brazilian Cohort, 2007-2014

**Variables**	**Subsequent cases with diagnostic date >2 months (N=227)**		**Subsequent cases with diagnostic date >6 months (N=163)**		**Subsequent cases with diagnostic date >12 months (N=114)**	
	**OR[1] (95%CI)**	**OR_{adj}[2,3] (95%CI)**	**OR[1] (95%CI)**	**OR_{adj}[2,3] (95%CI)**	**OR[1] (95%CI)**	**OR_{adj}[2,3] (95%CI)**
	**(N=20,553)**	**(N=13,350)**	**(N=20,486)**	**(N=13,306)**	**(N=20,428)**	**(N=13,272)**
**Household characteristics**						
Area of residence						
Urban	1.00		1.00		1.00	
Rural	1.02 (0.70-1.49)		0.99 (0.63-1.53)		0.97 (0.57-1.65)	
Per capita income						
≥0.25 minimum wage	1.00		1.00		1.00	
0 - 0.24 minimum wage	1.63 (0.96-2.77)		1.74 (0.93-3.27)		1.80 (0.86-3.78)	
0 minimum wage	1.84 (0.91-3.68)		1.90 (0.83-4.33)		0.87 (0.28-2.74)	
Household density						
0 - 0.99 inhab/room	1.00		1.00		1.00	
1.0 - 1.49 inhab/room	1.24 (0.83-1.85)		1.25 (0.77-2.03)		1.13 (0.62-2.06)	
≥1.5 inhab/room	1.37 (0.91-2.09)		1.72 (1.06-2.79)		2.15 (1.21-3.81)	
Housing construction material						
Bricks/cement	1.00		1.00		1.00	
Taipa/Wood/Others	1.00 (0.72-1.40)		1.12 (0.75-1.68)		1.05 (0.66-1.70)	
Water supply						
Public network (tap water)	1.00		1.00		1.00	
Well/natural source/Others (cisterna or other not described)	0.90 (0.64-1.27)		0.96 (0.64-1.44)		0.89 (0.56-1.43)	
Waste disposal system						
Public network	1.00		1.00		1.00	

© 2020 Teixeira CSS et al. *JAMA Dermatology.*

Homemade or Septic tank	1.20 (0.81-1.78)		1.38 (0.89-2.16)		1.17 (0.68-2.01)	
Ditch/Others	1.34 (0.80-2.23)		1.47 (0.83-2.61)		1.60 (0.81-3.16)	
Electricity supply						
Electricity with counter	1.00		1.00		1.00	
Electricity without counter/ Gas/Candlelight/Others	1.03 (0.70-1.53)		1.11 (0.71-1.75)		1.40 (0.84-2.34)	
Garbage disposal						
Public collection system	1.00		1.00		1.00	
Burned/buried/Outdoor disposal/ Others	0.83 (0.58-1.21)		0.77 (0.50-1.19)		0.99 (0.60-1.64)	
**Clinical characteristics of the primary case**						
WHO operation classification						
Paucibacillary	1.00	1.00	1.00	1.00	1.00	1.00
Multibacillary	1.47 (1.05-2.06)	1.59 (1.01-2.49)	1.74 (1.17-2.60)	2.36 (1.33-4.17)	2.03 (1.25-3.30)	3.49 (1.72-7.08)
Physical disability at the diagnosis						
Grade 0	1.00		1.00		1.00	
Grade 1	0.87 (0.56-1.34)		0.93 (0.56-1.56)		1.34 (0.76-2.39)	
Grade 2	1.10 (0.54-2.25)		1.58 (0.73-3.42)		2.15 (0.90-5.13)	
Reaction episode						
No reaction	1.00		1.00		1.00	
Reaction type 1	1.32 (0.79-2.22)		1.40 (0.76-2.58)		2.19 (1.13-4.23)	
Reaction type 2	1.73 (0.69-4.33)		2.07 (0.72-5.98)		2.87 (0.90-9.19)	
Reaction type 1 + 2	2.37 (0.61-9.22)		4.04 (0.96-17.10)		7.09 (1.55-32.52)	
**Individual characteristics of the contacts**						
Sex						
Female	1.00	1.00	1.00	1.00	1.00	1.00

© 2020 Teixeira CSS et al. *JAMA Dermatology.*

Male	1.35 (0.99-1.83)	1.73 (1.16-2.60)	1.27 (0.89-1.81)	1.86 (1.14-3.03)	1.36 (0.89-2.09)	1.72 (0.98-3.04)
Age						
<5 years	1.00	1.00	1.00	1.00	1.00	1.00
5 - 9 years	1.06 (0.74-1.53)	0.92 (0.58-1.47)	0.86 (0.57-1.30)	0.79 (0.47-1.35)	0.86 (0.53-1.39)	0.68 (0.37-1.24)
10 - 14 years	0.84 (0.56-0.25)	0.56 (0.33-0.96)	0.59 (0.37-0.95)	0.31 (0.16-0.61)	0.55 (0.31-0.97)	0.27 (0.12-0.60)
Race/Ethnicity						
White	1.00		1.00		1.00	
Not White	1.38 (0.87-2.17)		1.59 (0.92-2.76)		2.15 (1.04-4.42)	
Schooling (head of family)						
High school/College	1.00		1.00		1.00	
Elementary/Middle school (4 - 9 years of formal education)	0.63 (0.39-1.00)		0.55 (0.33-0.93)		0.45 (0.24-0.83)	
Elementary school (< 4 years of formal education)	0.84 (0.53-1.35)		0.77 (0.46-1.30)		0.64 (0.34-1.18)	
Illiterate/preschool	0.29 (0.13-0.65)		0.07 (0.02-0.33)		0.09 (0.02-0.44)	
Work condition (head of family)						
Employed	1.00		1.00		1.00	
Unemployed but currently studying	0.90 (0.59-1.37)		1.00 (0.62-1.61)		0.93 (0.52-1.63)	
Unemployed	0.87 (0.54-1.40)		0.76 (0.42-1.35)		0.59 (0.28-1.23)	

[1]Univariate multilevel logistic regression model accounting for household and state level random effects.

[2]Final model of multilevel logistic regression accounting for household and state level random effects with a priori adjustment for operational classification of the primary case, and sex and age of the contact and exclusion of individuals with missing data.

[3]For all the tests and for inclusion of the variables in the final model was used a significance level of 5%. Multivariate models were created using a backward selection approach and evaluated using the Akaike Information Criterion. The goodness of fit of the final model was also assessed.
